# Association of Wild-Type TP53 with Downregulation of Lovastatin Sensitivity in Human Non-Small Cell Lung Cancer Cells

**DOI:** 10.3390/cimb46090604

**Published:** 2024-09-13

**Authors:** Yu-Yao Chang, Tsung-Ying Yang, Gwo-Tarng Sheu

**Affiliations:** 1 Institute of Medicine, Chung Shan Medical University, Taichung 402, Taiwan; 177176@cch.org.tw; 2 Division of Colon and Rectal Surgery, Department of Surgery, Changhua Christian Hospital, 135 Nanhsiao Street, Changhua 500, Taiwan; 3 Department of Chest Medicine, Taichung Veterans General Hospital, No. 1650, Sect. 4, Taiwan Boulevard, Taichung 407, Taiwan; 4 Department of Life Sciences, National Chung Hsing University, No. 145, Xingda Rd., South Dist., Taichung 402, Taiwan; 5 Department of Medical Oncology and Chest Medicine, Chung Shan Medical University Hospital, No. 110, Sec. 1, Jianguo N. Road, Taichung 402, Taiwan

**Keywords:** statins, lung cancer, TP53, sensitivity, HMG CoA reductase

## Abstract

Statins inhibit 3-hydroxy-3-methylglutaryl-CoA reductase (HMGCR), the rate-limiting enzyme of the mevalonate pathway, and reduce cholesterol synthesis. They also have been demonstrated to improve prognosis in patients with various cancers, suggesting a potential anti-cancer effect of statins. However, there is no consensus on the molecular targets of statins for their anti-cancer effects. Docetaxel (DOC) is a microtubule-stabilizing agent currently used as a chemotherapeutic drug in several cancers, including lung cancer. Interestingly, the anti-cancer effects of either drug that are related to abnormal or wild-type TP53 gene have been implied. Therefore, the drug sensitivity of DOC and lovastatin in human lung cancer cells was evaluated. We found that H1355 (mutant TP53-E285K), CL1 (mutant TP53-R248W), and H1299 (TP53-null) human non-small cell lung cancer cells were more sensitive to lovastatin than A549 and H460 cells expressing wild-type TP53. Conversely, A549 and H460 cells showed higher sensitivity to DOC than H1299 and CL1 cells, as demonstrated by the MTT assay. When endogenous TP53 activity was inhibited by pifithrin-α in A549 and H460 cells, lovastatin sensitivities significantly increased, and cancer cell viabilities markedly reduced. These results indicate that TP53 status is associated with the anti-cancer effect of statins in human lung cancer cells. Mutated or null TP53 status is correlated with higher statin sensitivity. Furthermore, DOC-resistant H1299 (H1299/D8) cells showed significant sensitivity to lovastatin treatment compared to DOC-resistant A549 (A549/D16) cells, indicating a potential application of statins/chemotherapy combination therapy to control wild-type and abnormal TP53-containing human lung tumors.

## 1. Introduction

The statin family of drugs targets HMG-CoA reductase (HMGCR), the rate-limiting enzyme of the mevalonate (MVA) pathway, and has been successfully used in the treatment of hypercholesterolemia for the past four decades. Lovastatin was approved by the FDA in 1987 as the first commercial statin [1]. Despite having identical working mechanisms and similar effects on cholesterol compositions, statins can be partitioned into one of two classes: type I are fungal-derived statins (lovastatin, pravastatin, simvastatin); and type II are synthetically-derived statins (fluvastatin, cerivastatin, atorvastatin, rosuvastatin, pitavastatin). Type I statins hold similar structural homology to mevastatin, the first statin to be developed [2]. Although type II statins keep the HMG-CoA-like lactone moiety for binding, these molecules are fully synthetic inhibitors of HMG-CoA reductase and display highly varied pharmacokinetic characters, including differences in metabolism, excretion, half-lives, bioavailability, dosing times, and lipophilicity [3]. Meanwhile, based on their efficacy in reducing low-density lipoprotein (LDL) levels, statins have been classified in three categories. First-generation statins including lovastatin, pravastatin and fluvastatin, whereas the second generation including simvastatin and atorvastatin. Rosuvastatin and pitavastatin are the third-generation statins. According to their solubility, statins are also separated as hydrophilic or lipophilic compounds. The major lipophilic statins (simvastatin, fluvastatin, lovastatin, pitavastatin, and atorvastatin) can smoothly pass into the membranes profoundly where they interact with the neighboring acyl chains. By contrast, hydrophilic statins (pravastatin and rosuvastatin) interacting with the polar exterior of the membrane and need protein carriers to enter the cell to impede the HMG-CoA reductase enzyme [4,5]. The most noticed side effects of statins are myotoxicity and rhabdomyolysis, which are believed to be related with the accompanying illness, including hepatic insufficiency, cholestasis or renal diseases [3].

According to a recent report from a meta-analysis, the use of statins was associated with a lower overall mortality of colon cancer, as well as a lower cancer-specific mortality of colon cancer than non-users [6]. The correlation of statin administration with clinical outcomes in ovarian cancer (OC) has been calculated by a comprehensive meta-analysis, and the results showed that the use of statins markedly ameliorated the overall survival (OS) time and OC-specific survival time, especially the OS time in patients with serous OC and endometrioid OC. In addition, the survival rate was higher in patients who used statins following OC diagnosis [7]. Whether statins administrated after breast cancer diagnosis can reduce the risk of specific or all-cause mortality in a large cohort of breast cancer patients has also been reported, and there was some evidence of reduced mortality in statin users after breast cancer diagnosis [8]. Based on the source of UK Clinical Practice Research Datalink, 3638 lung cancer patients had been administered statin before and after diagnosis, and there was some evidence that statin use after diagnosis was associated with reduced lung cancer-specific mortality [9]. Surprisingly, it has a non-significant effect on urinary bladder cancer (UBC) risk among statin users when compared to non-users, suggesting there was no statin effect on UBC incidence and overall prognosis [10]. The anti-cancer effect of statins correlated with the prognosis in various cancers is still debatable from epidemiologic evidence.

People with non-small cell lung cancer (NSCLC) can be handled with surgery, chemotherapy, radiation therapy, targeted therapy, immunotherapy, or a combination of these treatments. Generally, NSCLC is diagnosed at an advanced stage in a majority of patients for whom systemic therapy remains the basis of treatment [11]. The poor efficacy and noticeable toxicity of chemotherapy have caused great pessimism for many years regarding this approach, as only a small positive impact on survival rates was observed. Therefore, there is growing interest in natural compounds used in earlier stages of the disease when combined with other anti-lung cancer therapies [12].

It has been reported that a combination of atorvastatin (Type II) with carboplatin reduces tumor growth and enhances survival rates more than carboplatin alone, as demonstrated in an experimental xenograft mice model with A549 cells [13]. These data indicate the potential for combining statins with chemotherapy. Docetaxel (DOC) stimulates microtubule assembly and stabilizes the polymers by binding to β-tubulin and against depolymerization, thereby inhibiting microtubule dynamics [14]. As a result, mitotic progression is restricted. Currently, statins have no reported effects on the activity of the mitotic spindle. Interestingly, the molecular mechanisms evoked in response to statins or docetaxel have been investigated previously in other cell cultures and animal models. The effects of combining lovastatin and paclitaxel in the human leukemia cell lines (K562 and HL-60) have been examined, and a synergistic interaction was observed [15]. The combination of DOC with statins has been tested in the human gastric HGT-1 cancer cell line, and the results showed that the association of lovastatin and DOC provided a more than additive apoptotic response [16], whereas lovastatin and paclitaxel combination did not show any increase in cytotoxicity in anaplastic thyroid carcinoma cells [17].

The *TP53* gene, which encodes the TP53 protein transcription factor, is able to trigger a multitude of tumor-suppressive effects in response to a wide variety of cellular stress signals. *TP53* mutations are present in approximately 45% of lung adenocarcinomas, and they are correlated. Therefore, highlighting these mutants became attractive targets for lung cancer therapy. Turrell et al. have reported a *TP53*^R270H^-specific sensitivity to simvastatin (Type I) in murine lung tumors and demonstrated that the transcriptional activities associated with this statin sensitivity were also present in human lung tumors [18]. In particular, R270H mutant tumors revealed enhanced MVA pathway gene expression, and R270H murine cell lines showed a strong association with this transcriptional signature [18]. Indeed, MVA pathway inhibition through simvastatin treatment induced vigorous cell cycle arrest and apoptotic responses in R270H lung tumors but had no significant impact on *TP53*-null or R172H murine lung tumors [18]. Further determining whether mutant *TP53* status and *TP53*-null are associated with this benefit could help patients benefit from statin repurposing for human lung cancer therapy. In breast cancer studies, the MVA pathway is both necessary and sufficient to maintain the malignant state of breast cancer cells in three-dimensional (3D) cultures. Knockdown of mutant *TP53* from breast cancer cells used in the 3D culture model significantly downregulates the MVA pathway, revealing a potential mechanism by which mutant *TP53* increases expression of the genes in the MVA pathway [19].

At a clinical level, inhibition of the MVA pathway by statins, either alone or in combination with other therapies, may offer a novel and much-needed therapeutic option for tumors bearing abnormal *TP53*. In this study, we have applied human NSCLC cell lines containing wild-type, mutated, or null *TP53* to test whether human lung cancer cells have different lovastatin sensitivity and the role of *TP53* in this event. Furthermore, the effect of the combination of DOC and lovastatin also have been investigated in chemoresistant cells.

## 2. Materials and Methods

### 2.1. Cell Culture

Human lung cancer A549, H460, H1299, and H1355 cells were obtained from the American Type Culture Collection (ATCC, Manassas, VA, USA). CL1-0 and CL1-5 cells were kindly provided by Dr. P.-C. Yang (Department of Internal Medicine, National Taiwan University Hospital, Taipei, Taiwan) [20]. The H460, A549, and H1299 cancer cell lines were maintained in DMEM (Gibco, Life Technologies, Carlsbad, CA, USA). The H1355, CL1-0, and CL1-5 (derived from CL1-0 with epithelial-mesenchymal transition phenotype) lung cancer cell lines were maintained in RPMI-1640 medium containing 10% fetal bovine serum supplemented with penicillin (100 U/mL) and streptomycin (100 mg/mL) (Gibco). Cells were grown in a 37 °C humidified incubator with 5% CO_2_. CL1 is harboring a mutated TP53 gene (R248W), H1355 with a mutated *TP53* gene (P72R, E285K), A549 and H460 harboring wild-type *TP53* (wt-*TP53*) and *TP53*-null for H1299 cells. The DOC-resistant A549 and H1299 sublines were established from parental cells in a stepwise manner by exposure to increasing concentrations of DOC as previously described [21]. Initially, the low cellular density of A549 or H1299 cells was seeded onto a 10 cm Petri dish, and 0.5 nM DOC was added to kill most of the cells. Until the surviving cells grew to an obvious colony, the colony was picked and transferred into a 48-well plate. The selected colony was amplified in the presence of 0.5 nM DOC until confluence before the drug dose increased in multiples of two for the next round of selection. These drug-resistant sublines were proliferating in the indicated DOC concentration.

### 2.2. MTT Cell Viability Assay

The cell lines were cultured in 96-well flat-bottomed microtiter plates, with cells reaching the exponential growth phase before drug treatment. Following 48 h of incubation with each drug, the in vitro cell viability induced by DOC or lovastatin was determined by MTT assay (at 570 nm) using an ELISA plate reader (Molecular Devices SPECTRA max 340 PC) as previously described [22]. Cell viability was expressed as a percentage of cells (% of control) without DOC or lovastatin (Sigma-Aldrich, St. Louis, MO, USA) treatment, and the mean values were calculated from three independent experiments. Cell images were captured using an inverted microscope. The TP53 inhibitor pifithrin-α (PFTα, Sigma-Aldrich), which inhibits *TP53*-mediated apoptosis and *TP53*-dependent gene transcription, was applied to pretreat cancer cells, followed by DOC or lovastatin. Dimethyl sulfoxide (DMSO) as a solvent was obtained from Gibco.

### 2.3. Statistical Analysis

T-test analysis was performed using SPSS software (Version 13.0 SPSS Inc., Chicago, IL, USA) for statistical analysis. A value of *p* < 0.05 was considered to be statistically significant.

## 3. Results

### 3.1. Higher Lovastatin Sensitivity of Abnormal TP53-Containing Lung Cancer Cells than wt-TP53 Cells

**Figure 1:**
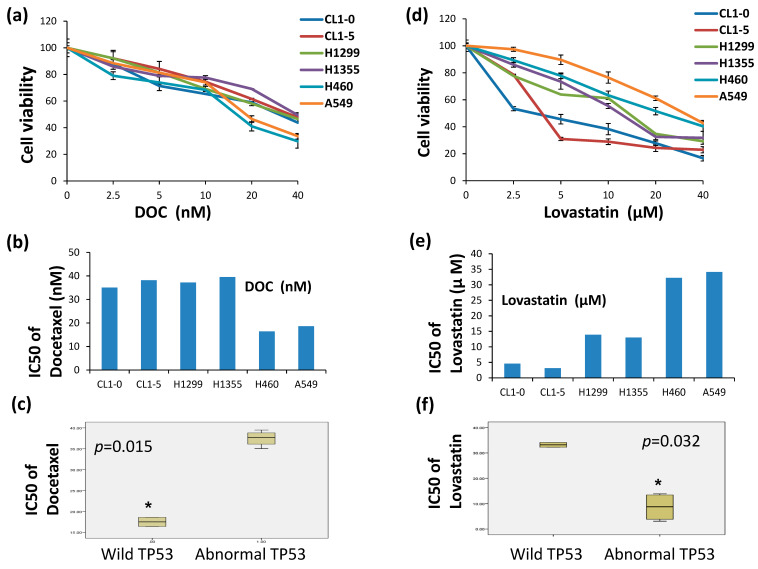
Determination of the DOC and lovastatin sensitivities in lung cancer cells by MTT assay. To evaluate the sensitivity of DOC and lovastatin in lung cancer cells with different TP53 statuses, varied concentrations of DOC (**a**) (2.5 to 40 nM) and (**d**) lovastatin (2.5 to 40 μM) were applied on A549, H460, H1299, H1355, CL1-0, and CL1-5 cells. (**b**) The half-maximal inhibitory concentration (IC50) of each cell line to DOC and (**e**) lovastatin were determined accordingly. IC50 for each cell line to (**c**) Either DOC or (**f**) lovastatin sensitivity were determined and statistically analyzed with the TP53 status, respectively. * A value of *p* < 0.05 was considered to be statistically significant.

To compare lovastatin and docetaxel (DOC) sensitivity in cells containing the different statuses of the *TP53* gene, A549 and H460 cells (wt-*TP53*), H1299 cells (null-*TP53*), H1355 (*TP53*^P72R, E285K^), CL1-0 and CL1-5 cells (*TP53*^R248W^) were used. Varied concentrations of DOC (2.5 to 40 nM) and lovastatin (2.5 to 40 μM) were applied to A549, H460, H1299, H1355, CL1-0, and CL1-5 cells and cell viability was measured by MTT assay. The half-maximal inhibitory concentration (IC50) for each cell line to DOC was calculated and listed in (Figure 1b,e). The data showed that A549 and H460 cells were more sensitive to DOC than H1299, H1355, CL1-0 and CL1-5 cells (Figure 1a). When cells were treated with lovastatin (Figure 1d), A549 and H460 cells were less sensitive to lovastatin than other cells (Figure 1d). The half-maximal inhibitory concentration (IC50) of each cell line to DOC was 35, 38, 37, and 39 nM accordingly (Figure 1b). The IC50 for each cell line to lovastatin was 5, 3, 14, 13, 32, and 34 μM accordingly (Figure 1e). The IC50 values were determined and statistically analyzed with the *TP53* status (Figure 1c,f), respectively. The results showed a significant decrease in IC50 of docetaxel (DOC) in wt-*TP53* cells (*p* < 0.015) and lower IC50 of lovastatin in abnormal *TP53*-containing cells (*p* < 0.032).

### 3.2. Inhibition of wt-TP53 Protein by Pifithrin-α (PFTα) Enhances Lovastatin Sensitivity but Reduces Docetaxel Sensitivity in Human Lung Cancer Cells

**Figure 2:**
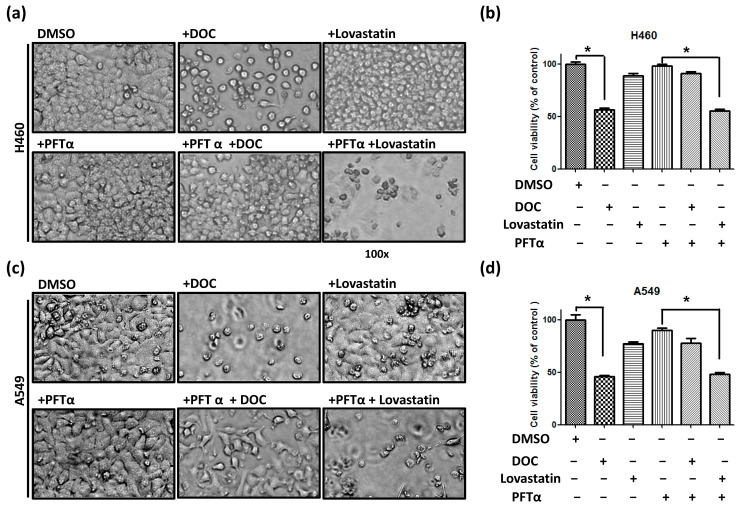
Inhibition of wt-TP53 protein by PFTα followed by DOC or lovastatin to measure drug sensitivity of lung cancer cells. (**a**) H460 cells were pretreated with DMSO or PFTα (10 μM) for 48 h, then treated either with DOC (20 nM) or lovastatin (10 μM) for another 48 h. Similar treatments were applied to (**c**) A549 cells, and the images of the cells were taken with 100× magnification using a light microscope. The viability of (**b**) H460 cells and (**d**) A549 cells were calculated, followed by an MTT assay. * Statistically significant.

Considering the potential role of wt-*TP53* in regulating drug sensitivity, human lung cancer cells were pretreated with PFTα (10 μM) for 48 h in H460 and A549 cells. Subsequently, cells were treated with DOC (20 nM) or lovastatin (10 μM) for 48 h followed by MTT assay. The cell images of H460 (Figure 2a) and A549 (Figure 2c) cells were obtained accordingly, and the cell viability of H460 (Figure 2b) and A549 (Figure 2d) cells were analyzed. When cells were treated with PFTα alone, no significant inhibitory effect was detected when compared with control cells (DMSO). The data showed that inhibition of wt-TP53 resulted in decreased DOC sensitivity. When both cell lines were treated with lovastatin and PFTα, the cell viabilities were significantly reduced compared with cells treated with PFTα alone. These results demonstrated that inhibition of wt-*TP53* increased lovastatin sensitivity in lung cancer cells harboring the wt-*TP53* gene. Therefore, our data suggest that the loss of functional TP53 protein may result in higher statin sensitivity in human lung cancer cells.

### 3.3. Comparison of the Lovastatin Efficacy in Parental Lung Cancer A549 and H1299 Cells and Their DOC-Resistant Sublines

**Figure 3:**
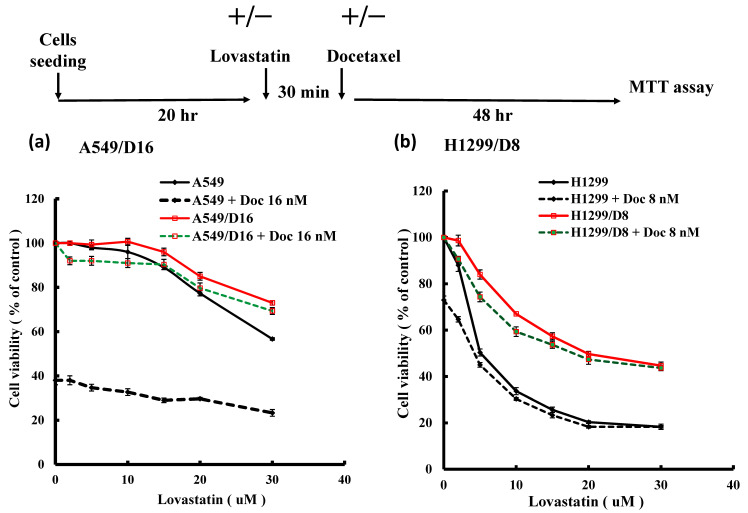
Lovastatin sensitivities of DOC-resistant A549 and H1299 sublines. (**a**) A549 cells were pretreated either with varied concentrations of lovastatin alone for 30 min or in combination with DOC (16 nM) for an additional 48 h. Similar treatments were applied to A549/D16 cells followed by MTT assay (**b**) H1299 cells and H299/D8 cells were treated either with varied concentrations of lovastatin alone or combined with DOC (8 nM) for 48 h followed by MTT assay.

We were interested in investigating whether lovastatin has any growth inhibition function on chemoresistant lung cancer cells. The chemoresistant A549/D16 and H1299/D8 cells were established previously [21]. Therefore, the sensitivity of DOC and lovastatin on lung cancer cells and DOC-resistant sublines was characterized by MTT viability assay. When A549 cells were treated with lovastatin, high concentrations of lovastatin (20, 30 µM) showed a moderate suppression of viability (Figure 3a, black-solid line). To confirm the sensitivity of A549 cells to DOC (Figure 3a, black-dotted line), cells were treated with 16 nM DOC without lovastatin, resulting in approximately 40% viability. When A549 cells were exposed to increased lovastatin concentrations and DOC (16 nM), minimal inhibition of cell viability was observed (Figure 3a, black-dotted line). Chemoresistant A549/D16 cells were confirmed when cells were exposed to DOC (16 nM), as shown in Figure 3a (red-solid line) with 100% viability. However, only high concentrations of lovastatin combined with DOC resulted in reduced viability in A549/D16 cells (Figure 3a, red-solid line). Since A549/D16 cells did not respond to 16 nM of DOC treatment, those cells treated with lovastatin alone (Figure 3a, red-solid line) showed a similar response as when both drugs were combined (Figure 3a, green-dotted line). A more pronounced response was detected when H1299 and DOC-resistant H1299 cells (H1299/D8) were used under similar experimental conditions (Figure 3b). When comparing lovastatin sensitivity between A549/D16 and H1299/D8 cells or between A549 and H1299 cells, both H1299/D8 and H1299 cells exhibited higher lovastatin sensitivity than A549/D16 and A549 cells, respectively. Although H1299/D8 cells showed significant sensitivity to lovastatin (Figure 3b, red-solid line), developed chemoresistance may reduce the growth inhibition effect of lovastatin when compared to their parental H1299 cells (Figure 3b, black-solid line).

### 3.4. Correlation between TP53 Status and Sensitivity to Lovastatin and Docetaxel (DOC) in Human Lung Cancer Cells

**Figure 4:**
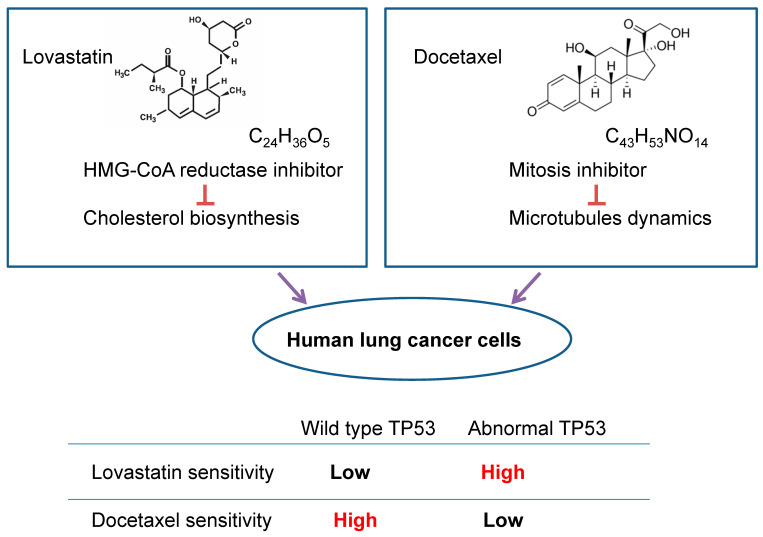
A simplified conclusion has been obtained and summarized.

Based on the data presented above, a general conclusion can be drawn and illustrated in Figure 4. Human lung cancer cells harboring abnormal *TP53* have higher lovastatin sensitivity than cells harboring wt-*TP53*. Conversely, human lung cancer cells with abnormal *TP53* have lower docetaxel sensitivity than cells harboring wt-*TP53*.

Human lung cancer cells harboring wt-*TP53* have higher DOC sensitivity than cells harboring abnormal *TP53.* Whereas, human lung cancer cells harboring abnormal *TP53* have higher lovastatin sensitivity than cells harboring wt-*TP53*. As discussed later, we hypothesized that cancer cells harboring abnormal TP53 expressing higher levels of MVA pathway genes result in higher statin sensitivity than cells with wt-TP53. Further, we have reported that DOC transactivated β-tubulin genes in H1299 cells but not in the A549 cells [23]. Therefore, it is possible that when more β-tubulins are produced in lung cancer cells harboring abnormal *TP53,* that may reduce DOC cytotoxicity markedly.

## 4. Discussion

The activities of statins have been extensively reviewed and summarized and have been found to reduce cell proliferation ability both in in vitro and in vivo animal models [24]. Previous studies using a population-based cancer data source in the United States have indicated that statin users are associated with improved survival among patients with stage IV NSCLC [25]. A meta-analysis also suggested that statins potentially enhanced the effects of tyrosine kinase inhibitors (TKIs) and chemotherapy on the overall survival of patients with NSCLC [26]. These findings suggest that statins may potentially enhance the anti-lung cancer effect. However, there is no consensus on the molecular targets of statins for their anti-lung cancer effects. In this study, we investigated whether statin sensitivity is dependent on the status of the tumor suppressor gene *TP53* in in vitro human lung cancer cells.

The *TP53* gene is known to be associated with the outcome of cancer treatment by regulating the transcription of downstream genes in response to various stressors. These induced genes include cell cycle checkpoints, DNA repair, metabolic homeostasis, autophagy, and apoptosis [27]. A critical investigation has shown that activation of endogenous *TP53* downregulates the MVA pathway in human SK-HEP-1 hepatocellular carcinoma cells, HCT116 colon cancer cells, and mouse embryonic fibroblasts (MEFs) [28]. Furthermore, *TP53*-null and *TP53* mutant cancer cells exhibit higher levels of MVA pathway gene expression compared to other cancer cell lines with wt-*TP53*. Moreover, in lung cancer patients with mutant *TP53*, statin users have better survival rates than non-statin users, while statins worsened the prognosis of those patients with wt-*TP53* significantly [29]. Mutation of *TP53* promotes cancer malignancy, and it has become a target of anti-cancer therapy [27].

In this report, we found that H1355, CL1 (mutant *TP53*), and H1299 (*TP53*-null) NSCLC cells were more sensitive to lovastatin than A549 and H460 cells expressing wt-TP53. Conversely, A549 and H460 cells exhibited higher sensitivity to docetaxel than H1299 and CL1 cells. When TP53 activities were inhibited by pifithrin-α in A549 and H460 cells, lovastatin sensitivities were significantly increased, and viabilities were reduced. These results indicate a *TP53*-associated anti-cancer effect of statins. We further tested whether statins reduce the viability of DOC-resistant lung cancer cells. Lovastatin reduced cell viability in a dose-dependent manner in DOC-resistant H1299 cells, and the addition of DOC had little antagonistic or additional effect. Other reports also provide evidence that DOC activates TP53 expression. A cis-sequence located at the *TP53* core promoter regulates p53 induction by DOC has been demonstrated [30]. It has been found that DOC was more sensitive in C4-2 and LNCaP prostate cancer cells that express wt-tp53 than DU145 cells that express mutant tp53 [31]. Interestingly, lovastatin’s ability to reduce the viability of DOC-resistant H1299 cells suggests for the first time that statins may overcome chemoresistant cells containing abnormal *TP53*.

Although our data indicate that different *TP53* statuses determine drug sensitivity to DOC and statins, other factors may also affect statin sensitivity. The limitation of this investigation is that we only used a simple in vitro cell viability assay to evaluate drug sensitivity in human lung cancer cells. Other factors have been discussed with transcriptome data from 14 NCI-60 cancer cell lines and their statin dose–response data [32]. Furthermore, an interesting report suggested that lung cancer A549 cells with high expression of thyroid transcription factor 1 (TTF-1) associated with low cholesterol were more vulnerable to simvastatin [33]. Therefore, TTF-1 has been considered as a putative sensitivity biomarker to statins in human lung cancer.

## 5. Conclusions

Combining non-tumor medicines with potential anti-cancer activities with conventional cancer therapy may present an opportunity to reduce costs and enhance patient outcomes. However, a limitation of cell line models is the lack of genetic diversity found in clinical tumors. Given the genetic heterogeneity of tumor cells, administering drugs that selectively target cells with different *TP53* statuses simultaneously may provide a novel strategy for treating certain subtypes of lung cancer.

## Data Availability

The data presented in this study are available on request from the corresponding author.
